# Characterization of antibodies elicited by XMRV infection and development of immunoassays useful for epidemiologic studies

**DOI:** 10.1186/1742-4690-7-68

**Published:** 2010-08-17

**Authors:** Xiaoxing Qiu, Priscilla Swanson, Ka-Cheung Luk, Bailin Tu, Francois Villinger, Jaydip Das Gupta, Robert H Silverman, Eric A Klein, Sushil Devare, Gerald Schochetman, John Hackett

**Affiliations:** 1 Infectious Diseases R&D, Abbott Diagnostics, 100 Abbott Park Rd, Abbott Park, IL, 60064, USA; 2 Department of Pathology, Emory University School of Medicine/Yerkes National Primate Research Center, 954 Gatewood Dr, Atlanta, GA, 30329, USA; 3 Department of Cancer Biology, Lerner Research Institute, Cleveland Clinic Foundation, 9500 Euclid Av, Cleveland, OH, 44195, USA; 4 Glickman Urological and Kidney Institute and LRI, Cleveland Clinic Foundation, 9500 Euclid Av, Cleveland, OH, 44195, USA

## Abstract

**Background:**

Xenotropic Murine Leukemia Virus-related Virus (XMRV) is a human gammaretrovirus recently identified in prostate cancer tissue and in lymphocytes of patients with chronic fatigue syndrome. To establish the etiologic role of XMRV infection in human disease requires large scale epidemiologic studies. Development of assays to detect XMRV-specific antibodies would greatly facilitate such studies. However, the nature and kinetics of the antibody response to XMRV infection have yet to be determined.

**Results:**

Three rhesus macaques were infected with XMRV to determine the dynamics of the antibody responses elicited by infection with XMRV. All macaques developed antibodies to XMRV during the second week of infection, and the predominant responses were to the envelope protein gp70, transmembrane protein p15E, and capsid protein p30. In general, antibody responses to gp70 and p15E appeared early with higher titers than to p30, especially in the early period of seroconversion. Antibodies to gp70, p15E and p30 persisted to 158 days and were substantially boosted by re-infection, thus, were identified as useful serologic markers. Three high-throughput prototype assays were developed using recombinant proteins to detect antibodies to these viral proteins. Both gp70 and p15E prototype assays demonstrated 100% sensitivity by detecting all Western blot (WB) positive serial bleeds from the XMRV-infected macaques and good specificity (99.5-99.9%) with blood donors. Seroconversion sensitivity and specificity of the p30 prototype assay were 92% and 99.4% respectively.

**Conclusions:**

This study provides the first demonstration of seroconversion patterns elicited by XMRV infection. The nature and kinetics of antibody responses to XMRV in primates were fully characterized. Moreover, key serologic markers useful for detection of XMRV infection were identified. Three prototype immunoassays were developed to detect XMRV-specific antibodies. These assays demonstrated good sensitivity and specificity; thus, they will facilitate large scale epidemiologic studies of XMRV infection in humans.

## Background

In 2006, a novel gammaretrovirus was identified in prostate cancer tissue using Virochip DNA microarray technology [1]. Cloning and sequencing of the gammaretrovirus revealed a close similarity to xenotropic murine leukemia viruses; thus, it was named Xenotropic Murine Leukemia Virus-related virus (XMRV). Initial screening using a nested reverse transcription-PCR (RT-PCR) assay found that XMRV was detectable in 10% (9/86) of tumor tissues from prostate cancer patients [1]. Subsequent studies revealed several important insights regarding XMRV: (a) infectious virus was produced from prostate cancer cell lines transfected with an XMRV genome derived from 2 cDNA clones, (b) the virus replicated in both prostate and non-prostate cell lines, (c) XMRV replication in the prostate cancer-derived cell line, DU145, is interferon sensitive, and (d) a human cell surface receptor required for infection with XMRV is xenotropic and polytropic retrovirus receptor 1 [2]. Finally, the characterization of integration sites in human prostate DNA provided unequivocal evidence for the capacity of XMRV to infect humans [3].

Indeed, the association between XMRV and prostate cancer was strengthened by recent studies demonstrating the presence of XMRV DNA as well as viral proteins in prostate cancers [4,5]. Using a quantitative PCR and immunohistochemistry, Schlaberg *et al*. found XMRV DNA in 6% and XMRV proteins in 23% of 233 tissues from prostate cancer patients [4]. Moreover, XMRV was found at a higher frequency in higher grade or more aggressive cancers [4]. Recently, XMRV has been also identified in 67% (68/101) of patients with chronic fatigue syndrome in the United States (U.S.) [6]. In contrast, another U.S. study reported the absence of XMRV in either CFS patients (0/50) or healthy controls (0/56) [7]. Furthermore, studies conducted in Northern Europe indicate a much lower or zero prevalence of XMRV in patients with prostate cancer [8,9] or with CFS [10-12]. Whether the discrepancies are due to differences in the geographic distribution of XMRV, technological differences between the assays used, clinical criteria for CFS patient selection, or genetic divergence of XMRV remains to be determined.

Gammaretroviruses are well-known pathogens causing leukemia, neurological disease, and immunodeficiency in mice, cats and some non-human primates [13,14]. As XMRV is the first reported human gammaretrovirus, its existence raises many questions with regard to the etiologic role of XMRV in prostate cancer and/or its association with CFS and other human diseases, its mode of transmission, and its geographic distribution. Addressing these questions requires epidemiologic studies in large cohorts of patients with prostate cancer, CFS and other types of diseases as well as in the general population. The relatively cumbersome nature of molecular technologies such as DNA microarrays, fluorescence in situ hybridization (FISH), reverse transcriptase polymerase chain reaction (RT-PCR) and PCR presents a significant challenge to executing such studies. Thus, high-throughput serologic assays that detect XMRV-specific antibodies would be of great value.

Since its discovery, XMRV has been partially characterized at the molecular and cellular level [1-3,15-18]. However, there is very limited information available regarding the viral life cycle, replication dynamics, tissue tropism, and the host immune response to XMRV infection. In fact, the nature and kinetics of antibody seroconversion induced by infection with XMRV have yet to be determined. This information is essential for the development of optimal XMRV-antibody screening assays.

To learn more about XMRV infection and potential serologic markers, rhesus macaques were experimentally infected with XMRV to establish an animal model for studying viral replication kinetics, tissue tropism, and the immune response [19]. The present study focuses on the characterization of antibody responses to XMRV infection and the identification of serologic markers useful for detection and screening. Furthermore, this study also describes the development of high-throughput prototype immunoassays for the detection of XMRV-specific antibodies.

## Results

### XMRV Viral Proteins

**Figure 1:**
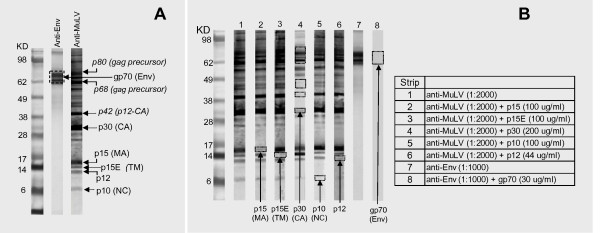
**XMRV viral proteins**. (A) XMRV viral proteins identified by WB analysis using goat polyclonal antibodies to Friend MuLV (anti-MuLV) at a 1:2000 dilution and to Env (gp69/71) of Rauscher-MuLV (anti-Env) at a 1:1000 dilution. Env, Envelope protein; TM, Transmembrane protein; MA, Matrix protein; CA, Capsid protein; and NC, Nucleocapsid protein. The *gag *precursor (p68/p80) and proteolysis intermediate (p12-CA) are italicized. (B) Competitive inhibition of anti-MuLV (Strips 2-6) and anti-Env (Strip 8) binding to native XMRV proteins on WB strips with recombinant XMRV proteins. Inhibitors of recombinant proteins and concentrations for specific strips are listed in the inserted table.

XMRV proteins were identified by Western blot (WB) analysis using goat polyclonal antibodies to Friend-MuLV (anti-MuLV pAb) and to envelope glycoprotein gp69/71 of Rauscher-MuLV (anti-Env pAb). Because XMRV shares >90% overall nucleotide sequence identity with known MuLVs, the anti-MuLV pAb detected all structural proteins of XMRV. The four mature core proteins derived from the *gag *gene, termed matrix (MA, p15), p12, capsid (CA, p30), and nucleocapsid (NC, p10) showed clearly resolvable bands on WB at molecular weights approximating the sequence prediction: MA at 15 kDa, p12 at 10 kDa, CA at 30 kDa and NC at 6 kDa (Figure 1A). In addition, the *gag *precursor (p68/p80) and proteolysis intermediate (p12-CA) were also detected. The transmembrane subunit (TM, p15E) of envelope protein showed a resolved band at 14 kDa on WB, although the sequence predicted molecular weight is 19.6 kDa (Figure 1A). The lower than predicted MW on SDS gel could be due to the elongated helical structure of TM protein [20]. The envelope protein gp70 was not clearly resolvable by the anti-MuLV pAb due to antibody binding to the *gag *precursor p68/p80 obscuring the region between 62 and 80 kDa. However, gp70 was clearly detected using the anti-Env pAb, showing diffuse doublet bands at ~70 kDa (Figure 1A).

The identity of XMRV structural proteins was further confirmed by competitive inhibition with recombinant XMRV proteins. *E. coli *expressed recombinant proteins, p15 (MA), p12, p30 (CA), p10 (NC) and p15E (TM), were used to competitively inhibit the anti-MuLV pAb binding to the corresponding native proteins on WB. As shown by Figure 1B (strips 2-6), band intensity of the native proteins decreased by 90-100% in the presence of the corresponding recombinant proteins confirming the banding positions for the native viral proteins: p15, p12, p30, p10 and p15E. The banding position of gp70 was confirmed by competitive inhibition of the anti-Env pAb with mammalian expressed gp70 protein as shown by Figure 1B (strip 8).

In summary, the data demonstrate that XMRV virions produced from prostate cancer cell line DU145 contain the four mature core proteins (p15, p12, p30, p10), and the two envelope proteins (gp70 and p15E). In addition, the WB method has the capacity to detect antibodies to all structural proteins of XMRV.

### Analysis of antibody response in XMRV-infected rhesus macaques

**Figure 2:**
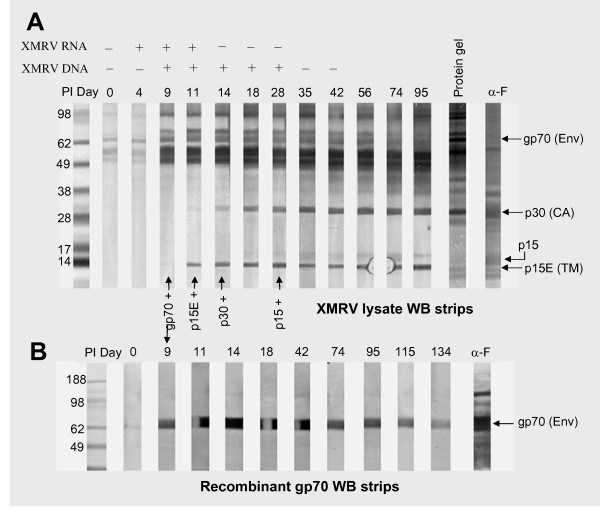
**XMRV seroconversion in RIl-10**. (A) Representative antibody responses detected by WB using native XMRV proteins (4 μg/strip) and (B) using mammalian expressed recombinant gp70 (~1.8 μg/strip). Plasma samples from macaque RIl-10 are listed on strips as days post inoculation (PI) with XMRV (0-134). Arrows indicate the first day that detectable reactivity was observed for specific viral proteins. XMRV viral RNA and proviral DNA results [19] are listed above the WB strips. The anti-MuLV pAb (α-F) was used as a positive control. The thin faint band at day 0 in Figure 2B most likely represents non-specific reactivity.

Serial bleeds from XMRV inoculated macaques were first analyzed by WB using native viral proteins. All three macaques developed XMRV-specific antibody responses during the second week post infection (PI). Figure 2A shows a representative antibody pattern (macaque RIl-10) during seroconversion of the XMRV-infected macaques and XMRV viral RNA and proviral DNA results [19]. The predominant antibody responses were to gp70, p15E and p30. The anti-gp70 response was first detected on day 9 PI, showing reactivity at 70 kDa. The anti-p15E response was first detected on day 11 PI and the anti-p30 response on day 14-18 PI in all three macaques. In addition, a weak antibody response to p15 (MA) was evident between days 28-35 PI in all macaques. Two macaques (RLq-10 and RYh-10) also developed weak and transient antibodies to p10 (NC) detectable from days 14 to 35 PI (data not shown).

The antibody response to gp70 was confirmed by WB using mammalian expressed recombinant gp70 antigen. As shown in Figure 2B, serial bleeds of RIl-10 from days 9 to 134 specifically bound to the recombinant antigen at 70 kDa. Specificity of the antibody responses to p15E and p30 was also confirmed by complete inhibition of binding to the native proteins in the presence of corresponding p15E or p30 recombinant proteins (data not shown). Of note, several major bands between 49 to 62 kDa (Figure 2A) that became substantially more intense on day 9 PI were subsequently confirmed to be human cellular proteins based on competitive inhibition studies utilizing uninfected DU145 cell lysate proteins (data not shown).

**Figure 3:**
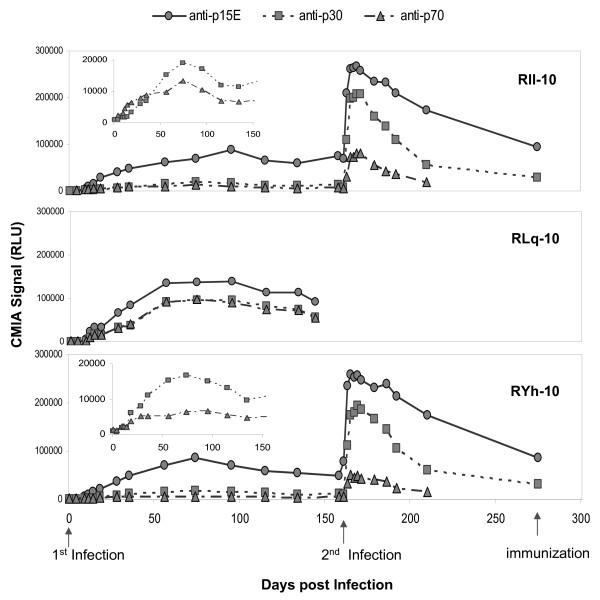
**Time course of XMRV-specific antibodies in macaques**. Detection of XMRV-specific antibodies in RIl-10, RYh-10 and RLq-10 using the recombinant protein (p15E, p70 or p30) based indirect chemiluminescent immunoassays (CMIAs). Macaque RLq-10 was sacrificed at 144 days. RLU, relative light units. Arrows indicate the XMRV-infection and immunization time points. The insets show anti-p30 and anti-p70 responses following 1^st ^infection on an expanded CMIA signal scale.

To determine the magnitude and the duration of the predominant antibody responses to XMRV, *E. coli *expressed recombinant antigens p15E, p70 and p30 were used to develop three indirect chemiluminescent immunoassays (CMIAs) on the automated ARCHITECT^® ^instrument system. Serial bleeds of the XMRV-infected macaques were analyzed by the indirect (anti-human IgG) CMIAs (Figure 3). All three macaques developed detectable antibody responses to p15E, p30 and p70 from days 9-18 PI (cutoff = signal ≥3 times of day 0 signal). Antibody titers increased to peak levels between days 74-95 and remained relatively stable to day 144 for RLq-10 (day of sacrifice) and day 158 for RIl-10 and RYh-10. After the second XMRV inoculation (day 158), the antibody responses were boosted substantially; the titers gradually decreased to basal levels and were maintained through day 275 PI.

Compared to the native viral protein-based WB, the indirect p15E assay was more sensitive. The assay detected all anti-p15E WB positive serial bleeds as well as the day 9 WB negative bleeds of all three macaques. The indirect p30 assay sensitivity and WB were similar; anti-p30 responses were detected on day 11 for RLq-10 and day 18 for RIl-10 and RYh-10. However, the indirect p70 assay was less sensitive than WB, initially detecting the day 11 bleed for RLq-10 and day 18 bleed for RIl-10 and RYh-10, 2-9 days later than the WB. Signals of the p70 assay were substantially lower as compared to signals of the p15E assay, perhaps due to incorrect folding of *E. coli *expressed p70 antigen which lacks glycosylation.

### Development of XMRV antibody assays

Although the three indirect p15E, p70 and p30 assays were sufficient to characterize antibody responses in the XMRV-infected macaques, they were not suitable for large scale epidemiologic studies in humans due to 3-5 fold higher background signals. Consequently, a direct assay format was used to improve detection specificity. In addition, the *E. coli *expressed p70 antigen was replaced with mammalian expressed gp70 recombinant protein in combination with signal amplification to improve assay sensitivity.

Using the *E coli *expressed recombinant proteins, p15E and p30 and mammalian expressed gp70, three direct CMIAs were developed for the automated ARCHITECT^® ^instrument system. All assays utilized a direct format where recombinant proteins were used for both capture and detection to form a double antigen sandwich with anti-p15E, anti-gp70 or anti-p30 antibodies. Specificity and sensitivity of the prototype assays were evaluated on blood donor samples (negative for other known bloodborne pathogens, presumed negative population) and the seropositive serial bleeds from the XMRV-infected macaques (positive population).

**Figure 4:**
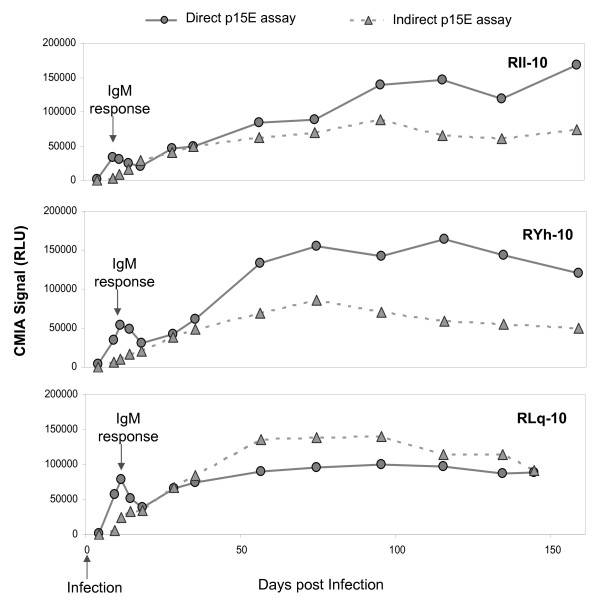
**Sensitivity comparison between the direct and indirect p15E CMIAs**. Comparison between direct and indirect p15E CMIAs for detection of XMRV p15E-specific antibodies in RIl-10, RYh-10 and RLq-10. The IgM response was confirmed using an anti-human IgM specific conjugate in the indirect assay format.

Figure 4 summarizes the results from sensitivity evaluation of both direct and indirect p15E CMIAs with 39 serial bleeds (days 4-144/158 PI) from XMRV-infected macaques, RIl-10, RLq-10 and RYh-10. Both the direct and indirect p15E assays detected 36 of 39 serial bleeds (days 9-144/158 PI); day 4 bleeds from each of the three macaques were negative in both assays. However, the direct p15E CMIA demonstrated better seroconversion sensitivity by generating significantly higher signals for the early IgM response (days 9-14 PI) in all three macaques, and better or equivalent sensitivity for the subsequent serial bleeds of RIl-10 and RYh-10.

**Figure 5:**
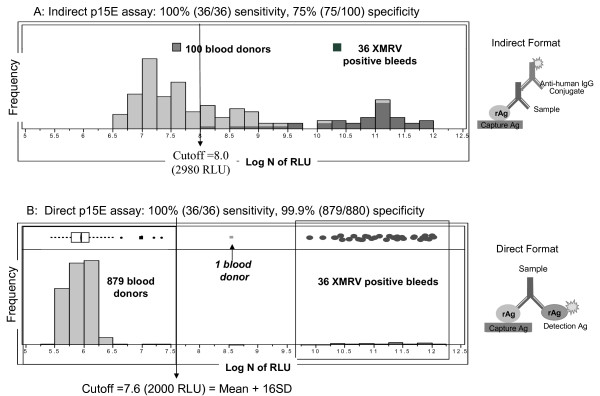
**Assay performance comparison between the direct and indirect p15E CMIAs**. (A) Signal distribution of the indirect format p15E CMIA (diagram shown) on 36 XMRV seropositive macaque bleeds and 100 blood donors. (B) Signal distribution of the direct format p15E CMIA (diagram shown) on 36 XMRV seropositive macaque bleeds and 880 blood donors. The box plot shows selected quantiles of continuous distributions (box), the median value (vertical line), the mean of 879 blood donors and 95% confidence interval (diamond). The 100 blood donors in (A) are a subset of the 880 blood donors in (B). Signals of the 36 XMRV seropositive bleeds were the same as plotted in Figure 4. Log N of RLU, natural log transformation of RLU.

The most significant advantage realized by utilization of the direct format assays is the improvement in specificity. This was demonstrated by a comparison between the direct and indirect p15E CMIAs on 100 blood donor samples. In the indirect p15E CMIA, signals of the blood donors were high (mean of 3275 RLU) with unacceptably broad distribution (standard deviation, SD, of 7019 RLU). The broad signal distribution resulted in poor separation between the negative population (100 blood donors) and the positive population (36 XMRV seropositive macaque bleeds). As shown in Figure 5A, based on a cutoff level set to detect all 36 XMRV seropositive bleeds, 25 of the 100 blood donors would be considered as false positive, resulting in an assay specificity of 75%. In contrast, the same 100 blood donor samples tested in the direct p15E CMIA had substantially reduced signals (mean of 446 RLU) and a far tighter distribution (SD of 38 RLU). An additional 780 blood donor samples were tested in direct p15E CMIA. Results obtained from the total 880 blood donor samples (set 1) showed a tight signal distribution with a mean of 383 RLU and SD of 100 RLU. Consequently, the 36 XMRV seropositive bleeds were clearly separated from the 880 negative blood donors (Figure 5B), resulting in 100% (36/36 XMRV macaque seropositive bleeds) sensitivity and markedly improved specificity of 99.9% (879/880). One donor sample (p81) was reactive (5059 RLU) by the direct p15E CMIA, but was negative by WB using viral lysate proteins (Additional file 1, section A1). To further evaluate assay specificity, specimens from 110 retrovirus infected humans (100 HIV-1, human immunodeficiency virus type I and 10 HTLV-I/II, human T-cell lymphotropic virus) were tested in the direct p15E CMIA. All were found to be non-reactive (data not shown).

**Figure 6:**
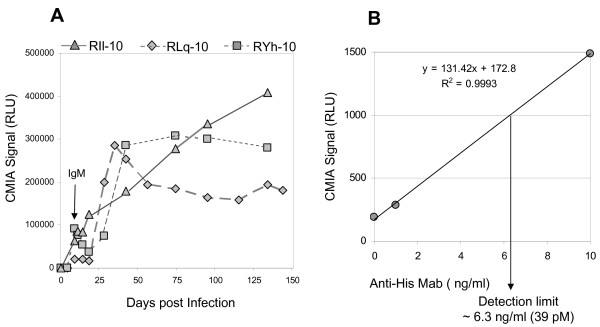
**Sensitivity evaluation of the direct gp70 CMIA**. (A) Detection of XMRV gp70-specific antibodies in RIl-10, RYh-10 and RLq-10 by the direct gp70 CMIA. All samples were diluted to 1:10 with negative human plasma prior to the testing. (B) Linear regression of signals at 10, 1 and 0 ng/ml of anti-His monoclonal antibody. Detection limit was determined based on the linear fitting equation with a cutoff value of 1000 RLU.

Sensitivity of the direct format gp70 CMIA was evaluated with 29 serial bleeds from the 3 XMRV-infected macaques (diluted to 1:10 with negative human plasma). As compared to the indirect p70 CMIA, detection sensitivity was greatly enhanced (Figure 6A). The direct gp70 CMIA demonstrated 100% sensitivity by detecting 1:10 dilutions of all 29 serial bleeds (days 9-134/144 PI) including 5 early bleeds (day 9 for RLq-10 and day 11 and 14 for both RIl-10 and RYh-10) that were not detected even when tested undiluted in the indirect p70 CMIA. The direct gp70 CMIA also exhibited good seroconversion sensitivity by detecting the early IgM response (days 9-14 PI) from all three macaques (Figure 6A). Since the recombinant gp70 protein contains a 6-histidine (His) tag sequence, analytical sensitivity of the direct assay could be determined using anti-His monoclonal antibody (anti-His Mab). Anti-His Mab was diluted in negative human plasma to concentrations of 100, 10 and 1 ng/ml and tested. As shown in Figure 6B, anti-His Mab could be detected at a level of 6.3 ng/ml or 39 pM. Assay sensitivity was also evaluated using end-point dilution analysis of anti-Env pAb. Using serial 2-fold dilutions in negative human plasma, the detection limit of the assay was estimated at 1:10,000 for this antiserum.

**Figure 7:**
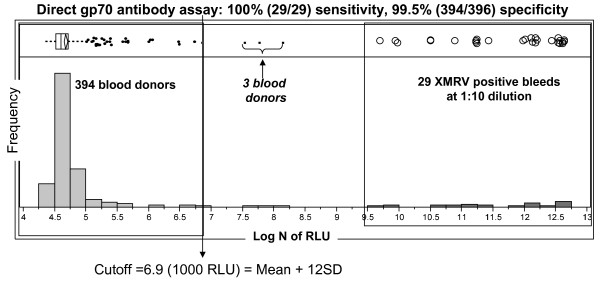
**Assay performance of the direct gp70 CMIA**. Signal distribution of the direct gp70 CMIA on 29 XMRV seropositive macaque bleeds (diluted 1:10) and 397 blood donors. The box plot shows selected quantiles of continuous distributions (box), the median value (vertical line), the mean of 394 blood donors and 95% confidence interval (diamond). Signals of the 29 XMRV seropositive bleeds at 1:10 dilution were the same as plotted in Figure 6A. Log N of RLU, natural log transformation of RLU.

Specificity of the direct gp70 CMIA was evaluated on a population of 397 blood donor samples (set 2). The signal distribution had a mean of 119 RLU and SD of 72 RLU. Three donor samples had signals above the assay cutoff of 1000 RLU; one (s44) had gp70 WB reactivity using recombinant gp70 antigen (Additional file 1, section A4). Excluding the WB reactive sample, specificity of the direct gp70 CMIA was estimated at 99.5% (394/396). The gp70 CMIA also showed substantial discrimination between the blood donor negative population and the 29 XMRV seropositive macaque bleeds even when diluted 1:10 (Figure 7).

Sensitivity of the direct p30 CMIA was initially evaluated using serial 10-fold dilutions of monoclonal antibody to MuLV p30 (anti-p30 Mab) or His (anti-His Mab). By linear regression, the detection limits were estimated to be 0.56 nM for the anti-p30 Mab and 1.18 nM for the anti-His Mab. As compared to the 39 pM detection limit of the direct gp70 CMIA for the anti-His Mab, the direct p30 CMIA is ~30-fold less sensitive. Seroconversion sensitivity was subsequently evaluated with 9 serial bleeds of RIl-10 from days 14 to 158 post the 1^st ^infection. Although the assay failed to detect the two early bleeds (days 14 and 18) that were positive by WB, it detected the remaining 7 bleeds. An additional 16 serial bleeds from RIl-10 and RYh-10 (days 5 to 52 post the 2^nd ^infection) were detected at a 1:10 dilution. Thus, the overall seroconversion sensitivity was 92% (23/25).

**Figure 8:**
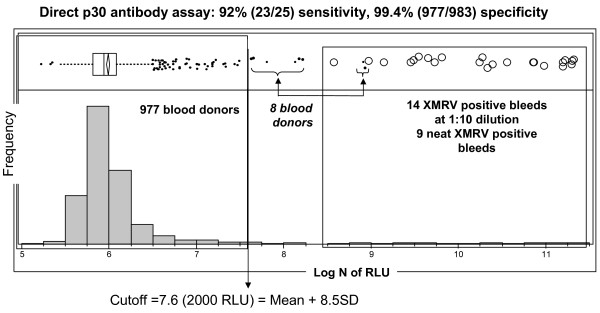
**Assay performance of the direct p30 CMIA**. Signal distribution of the direct p30 CMIA on XMRV seropositive macaque bleeds (9 neat and 14 diluted 1:10) and 985 blood donors. The box plot shows selected quantiles of continuous distributions (box), the median value (vertical line), the mean of 977 blood donors and 95% confidence interval (diamond). Log N of RLU, natural log transformation of RLU.

Specificity of the direct p30 CMIA was evaluated with a different set of 985 blood donor samples (set 3). Distribution of the assay values for the donor population had a mean of 420 RLU with SD of 195 RLU. The SD was 2-fold greater than the SD obtained using the direct p15E (SD = 100) and gp70 (SD = 72) CMIAs (Figure 8). Eight samples had values above the assay cutoff of 2000 RLU. Two of the 8 reactive donor samples (s176 and p43) had p30 WB reactivity (Additional file 1, section A3). Excluding the 2 WB reactive samples, specificity of the direct p30 CMIA was estimated at 99.4% (977/983). Due to broader distribution of the negative donor population and lower sensitivity in the early period of seroconversion, the direct p30 CMIA showed less discrimination between the negative donor and XMRV seropositive populations as compared to the direct p15E and gp70 CMIAs (Figure 8).

The 12 blood donor samples that were initially reactive in either the direct p15E, gp70 or p30 assay were re-tested in all the three direct CMIAs. Results are summarized in Table 1. In contrast to antibody responses in the XMRV infected primates that had high reactivity to all 3 proteins (S/CO ranges: 10-82 for p15E, 15-292 for gp70, 2.5-49 for p30), the blood donors showed low levels of detectable antibodies (S/CO < 3.7) and reactivity to only a single protein (either p15E, gp70 or p30) (Table 1). Based on WB analysis with viral lysate, two p30 CMIA reactive donor samples had anti-p30 reactivity (Additional file 1, section A3). A third donor had anti-gp70 reactivity on recombinant gp70 WB (Additional file 1, section A4). The lack of availability of PBMC or whole blood as well as plasma or serum from the 12 unlinked blood donors precluded attempts to confirm XMRV infection by PCR. Consequently, the 3 WB reactive blood donor samples were excluded from assay specificity calculations; the remaining 9 donor samples were designated as false positive for assay specificity calculations (Table 1).

**Table 1:** Serologic characterization of XMRV CMIA reactive blood donors

Donor ID	p15E CMIA	p30 CMIA	gp70 CMIA	WB		Designation for specificity calculation
	
	S/CO	S/CO	S/CO	Viral Lysate	gp70*	
p81	**2.5**	0.14	0.12	-	nt**	false positive

s44	0.2	0.16	**2.6**	-	gp70 band	excluded

s52	0.1	0.17	**2.2**	-	-	false positive

p52	0.1	0.15	**3.6**	-	-	false positive

p62	0.3	**1.0**	0.11	-	nt	false positive

s176	0.3	**1.1**	0.13	p30 band	nt	excluded

p43	0.2	**1.2**	0.12	p30 band	nt	excluded

s161	0.3	**1.7**	0.13	-	nt	false positive

s12	0.2	**1.7**	0.12	-	nt	false positive

s88	0.2	**1.8**	0.13	-	nt	false positive

s210	0.2	**3.7**	0.12	-	nt	false positive

p228	0.3	**3.7**	0.12	-	nt	false positive

* Mammalian expressed recombinant gp70

** not tested

### Antibody titers of the predominant responses in XMRV-infected macaques

To further characterize the predominant responses in XMRV-infected macaques, antibody titers of selected serial bleeds were determined using the 3 prototype direct CMIAs (Table 2). As expected, antibody titers correlated well with signals (RLU) of the CMIAs. After the initial infection, all 3 macaques showed similar titers for anti-gp70 and anti-p15E responses. However, RLq-10 had considerably lower titers for the anti-p30 response as compared to RIl-10 and RYh-10. Antibody titers to all three proteins were substantially boosted after the 2^nd ^inoculation with XMRV. The final immunization with a cocktail of recombinant XMRV proteins also boosted the anti-p15E titer by 10-fold and anti-p30 titer by 2.5 to 5-fold, but had no discernable impact on the anti-gp70 titer.

**Table 2:** Antibody titers of predominant responses in selected bleeds of XMRV-infected macaques and goat polyclonal antibodies to MuLV.

Sample	Days post XMRV Infection			Antibody Titers by ARCHITECT CMIAs		
	
	1^st ^ Infection	2^nd ^ Infection	Immuni- zation	Anti-gp70	Anti-p15E	Anti-p30
**RIl-10**	42			800	40	2
	
	134			3,200	160	10
	
	167	9		128,000	12,800	640
	
	210	52		32,000	800	80
	
	291	133	16	16,000	8,000	4,000

**RYh-10**	42			1,600	80	10
	
	134			1,600	80	10
	
	167	9		32,000	6,400	640
	
	210	52		8,000	400	80
	
	291	133	16	4,000	4,000	2,000

**RLq-10**	42			800	80	1
	
	134			800	160	1

**Anti-MuLV pAb**				16,000	32,000	64,000

**Anti-Env pAb**				10,000		

**Anti-His Mab**				0.039 nM		1.18 nM

**Anti-p30 Mab**						0.56 nM

Antibody titers were determined by the three direct gp70, p15E and p30 CMIAs on samples serially diluted with negative normal human plasma, and expressed as the reciprocal of the bleed dilution giving a reactive result. For comparison, detection limits of the anti-His and anti-p30 monoclonal antibodies were also listed.

**Figure 9:**
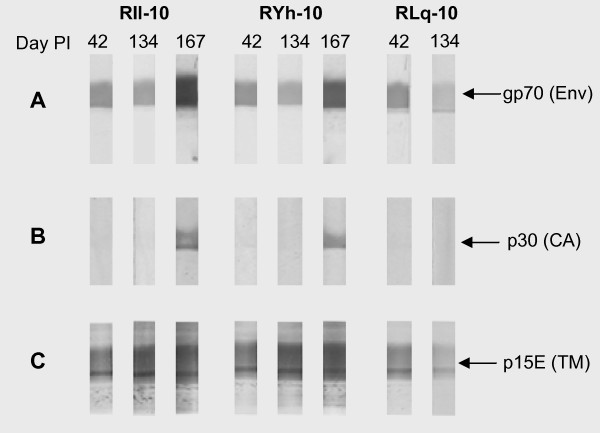
**Comparative reactivity for the predominant antibody responses**. Analysis of antibody responses in selected bleeds of RIl-10, RYh-10 and RLq-10 using WB strips prepared with recombinant proteins (90 pmole/protein): A, gp70 (4.4 μg/strip), B, p15E (4.1 μg/strip) and C, p30 (3.1 μg/strip). Plasma samples were listed on strips as days post inoculation (PI) with XMRV.

A comparison of the magnitude of antibody responses to each of 3 XMRV proteins is complicated by the difference in sensitivity between the three prototype assays. Moreover, the interpretation of WB results obtained using XMRV viral lysate may be compromised due to disparities in the quantity of each protein. In an effort to circumvent these issues, WB strips were prepared with recombinant proteins gp70, p15E and p30 at normalized concentrations of 90 pmole for each protein. WB reactivity of the day 42, 134, and 167 bleeds correlated with the CMIAs results. Notably, the anti-p15E response was as strong as the anti-gp70 response in all selected bleeds evaluated (Figure 9); both were present at days 42 and 134 and were boosted by re-infection (day 167). Anti-p30 reactivity was barely detectable at day 42 and 134 and was substantially boosted post-reinfection (day 167). These results confirmed that antibody responses to gp70 and p15E were dominant.

Based on the WB data obtained using XMRV viral lysate (Figure 1A), it was of interest to examine antibody titers of anti-MuLV pAb using the CMIAs. Anti-MuLV pAb contains high antibody titers to all three proteins, gp70, p15E and p30 (Table 2). Notably, the anti-p30 titer is ~100-fold higher than the titers present in the XMRV-infected macaques, presumably reflecting differences between antibody responses elicited by infection and immunization.

## Discussion

The primary objectives of the present study were to characterize the antibody response elicited by infection with XMRV and to develop high-throughput antibody assays suitable for large scale epidemiologic studies of XMRV infection. Since well-characterized XMRV antibody positive human specimens and seroconversion panels are currently unavailable, the utilization of a non-human primate model of XMRV infection provides a *bona fide *source of positive control sera and seroconversion samples useful for assay optimization and validation.

There is a paucity of information regarding the antibody response in humans following infection with XMRV. Several studies have reported detection of relatively low levels of neutralizing antibody or antibody cross-reactive to the surrogate envelope protein of Friend Spleen Focus Forming Virus (SFFV) in patients with prostate cancer, CFS, or blood donors [5,6,11]. Unfortunately, WB confirmation data is not available on these samples. Using recombinant based WB analysis of serum from prostate cancer patients and blood donors, Furuta *et al*. detected no antibody reactivity to XMRV envelope protein but occasional reactivity to XMRV *gag *protein [21]. Interpretation of these data is complicated by the lack of information regarding XMRV seroconversion patterns and suitable control reagents to determine assay sensitivity and specificity.

The present study provides the first demonstration of seroconversion patterns in primates following infection with XMRV and characterizes the nature and kinetics of the antibody response. All three experimentally infected macaques seroconverted to XMRV. The predominant antibody responses were directed against gp70, p15E and p30. Specific antibodies to gp70 and p15E appeared earlier during seroconversion and reached the highest titers. These characteristics are similar to the antibody responses elicited by MuLVs in mice [22-24]. Previous studies showed that both naturally occurring and vaccine induced responses to endogenous MuLVs were predominantly antibodies against gp70 and p15E [22-24]. Although antibody to p30 could be detected in certain mouse strains, the titers were lower relative to anti-gp70 or anti-p15E [23]. In addition, a primate model to assess potential risk of retroviral-mediated gene therapy also showed similar antibody responses to amphotropic MuLV [25]. In this study, 5 rhesus macaques experimentally infected with amphotropic MuLV developed antibodies to gp70 and p30 (anti-p15E response was not examined) that persisted through the last day tested (337-696 PI). Viral infection was also confirmed by PCR amplification of proviral DNA in peripheral blood mononuclear cells and lymph node tissue. However, the time course and titers of these specific antibody responses were not determined.

Notably, the well-characterized antibody responses in humans infected with HIV and HTLV are also primarily to the envelope proteins (HIV-gp120, HTLV-gp46), transmembrane proteins (HIV-gp41, HTLV-gp21), and core proteins of capsid and matrix (HIV-p24 and p17, HTLV-p24 and p19). Antibodies to envelope and transmembrane proteins were identified as the early and sustained serologic markers of infection [26-29]. These markers are the primary targets utilized by current third generation HIV and HTLV antibody assays as well as fourth generation HIV antigen/antibody combination assays for diagnostic testing and blood donor screening [30-33].

Taken together, the antibody responses to XMRV observed in this study are consistent with responses reported for other retroviruses. Thus, antibody responses to gp70, p15E and p30 represent potentially useful serologic markers for detection of XMRV infection.

Based on the identification of key serologic markers, prototype direct format CMIAs were developed using the recombinant proteins gp70, p15E or p30. The assays all showed good specificity (99.4-99.9%) with blood donor samples. Both gp70 and p15E prototype assays demonstrated 100% sensitivity by detecting all WB positive bleeds from XMRV-infected macaques. Seroconversion sensitivity of the p30 assay was slightly lower due to the combination of reduced analytic sensitivity and the delayed kinetics of the anti-p30 response. However, the p30 assay detects antibody to the core protein distinct from envelope proteins, thus, may still have value for confirmation of XMRV infection. Ideally, sensitivity and specificity of these prototype assays would be further validated using *bona fide *XMRV positive and negative human specimens once they become available.

Due to the high sequence homology, the assays described herein detect antibody responses not only to XMRV but also to other known MuLVs. Both the p15E and p30 prototype assays detected highly diluted (1:32,000 and 1:64,000) goat antibody to Friend MuLV. This is consistent with the high sequence homology of p15E (76%) and p30 (89%) proteins between XMRV and Friend MuLV. Interestingly, the gp70 assay was also able to detect the highly diluted antibodies to Friend MuLV (1:16,000) and Rauscher MuLV (1:10,000) despite lower sequence homology (59%) between the envelope proteins of these viruses. This suggests that the most conserved C-terminal domain of gp70 may represent the immunodominant region of the envelope protein.

In contrast to HIV and HTLV infection in humans that generally stimulate strong and sustained antibody responses, XMRV infection in macaques elicited detectable but less robust antibody responses. Re-infection with XMRV substantially boosted antibody titers; however, the titers decreased to a basal level by 110 days. The less vigorous antibody response may reflect a relatively low level of XMRV replication in macaques. This is consistent with the observation that only two (RIl-10 and RYh-10) of three chronically infected macaques had a detectable but low level plasma viremia (peak levels of 7,500 and ~2,000 copies/ml, respectively) after initial infection, and it was of short duration [19]. Moreover, although all three macaques had detectable provirus in PBMC, by 30 days PI XMRV was very difficult to detect in this compartment (Figure 2A) Of note, XMRV was detectable in various organs and tissues throughout a 9 month follow-up using PCR, FISH and immunohistochemistry [19]. Interestingly, a similar pattern of antibody response was also observed in HTLV-I infected pig-tailed macaques [34]. The boosted HTLV-I antibody responses decreased to a basal level approximately 60 days following the 2^nd ^HTLV-I infection but remained relatively stable over the next 8 months. Thus, the relatively weak and less sustained antibody responses observed in XMRV-infected macaques may not reflect what typically occurs in humans. Further investigations are needed to determine the level and duration of antibody responses in XMRV-infected humans.

## Conclusions

In summary, antibody responses elicited by XMRV-infection in the non-human primate model were fully characterized. The predominant responses to envelope protein gp70, transmembrane protein p15E, and capsid protein p30 were identified as useful serologic markers for detection of XMRV infection. Three high-throughput prototype antibody assays detecting these markers were also developed. The gp70 and p15E assays both demonstrated excellent sensitivity and specificity; thus, they will facilitate large-scale epidemiologic studies of XMRV infection in humans.

## Methods

### XMRV Virions

XMRV was cultured and purified by Advanced Biotechnologies, Inc. (ABI, Columbia, MD). Briefly, XMRV (VP62)-infected DU145 prostate cancer cells [2] obtained from the Cleveland Clinic (Cleveland, OH) were cultured in RPMI medium 1640 supplemented with 10% fetal bovine serum, 2 mM L-glutamine, 200 units penicillin G and 200 μg/ml streptomycin. Virus from the culture supernatants was purified by sucrose gradient centrifugation.

### Animals and virus inoculations

Three young adult rhesus macaques (>3 years old; >6 kg body weight) were selected from the Yerkes National Primate Research Center colony of Emory University. These included two males (RIl-10 and RLq-10) and one female (RYh-10). All three macaques were seronegative for antibodies to Simian immunodeficiency virus (SIV), Simian retrovirus (SRV) and Simian T-cell leukemia (STLV) and lacked cross-reactive antibodies to XMRV based on WB analysis using XMRV viral lysate.

After collection of baseline samples, each of the macaques was inoculated intravenously on day 0 with 10 ml of DU145 cell culture supernatant containing 3.6 × 10^5 ^TCID_50_/ml XMRV. One macaque (RLq-10) was sacrificed on day 144. To ensure persistent infections, 2 macaques (RIl-10 and RYh-10) were re-inoculated on day 158 with 3.6 × 10^6 ^TCID_50 _of purified XMRV virus. These 2 macaques were subsequently immunized on day 275 with 0.308 ml of recombinant XMRV proteins including p15 (2.3 μmole), p12 (1.1 μmole), p30 (3.1 μmole), p10 (2.9 μmole), p15E (3.8 μmole) and p70 (1.4 μmole) in incomplete Freund's adjuvant and sacrificed on day 291.

Blood was collected from each primate on days 0, 4, 5, 7, 9, 11, 14, 18, 21, 28, 35, 42, 56, 74, 95, 115, 134, 144, 158 post 1^st ^inoculation and on days 3, 5, 7, 9, 11, 13, 21, 28, 34, 52, 117 post 2^nd ^inoculation. Blood samples were obtained by venipuncture using tubes containing the anticoagulent EDTA and centrifuged at 250 × g for 10 minutes at room temperature. The plasma samples were collected in 0.5 ml aliquots and stored at -80°C.

### Human specimens and MuLV antibodies

A total of 2262 random blood donor specimens (1080 sera and 1182 plasma) were obtained from the Gulf Coast Regional Blood Center (Houston, TX). All samples were non-reactive for bloodborne infectious diseases on donor screening tests; these included: HBsAg, anti-HCV, anti-HBc, anti-HIV-1/HIV-2, HIV-1 NAT, HCV NAT, anti-HTLV-I/II, Syphilis, West Nile Virus, and Chagas. Plasma specimens from 100 HIV-1 seropositive Cameroonian blood donors collected in 2007 in accordance with local country regulations were provided by Drs. Lazare Kaptué (Université des Montagnes, Bangangté, Cameroon) and Lutz Gürtler (Max von Pettenkofer Institute, Ludwig-Maximilian Universität, Munich, Germany). Plasma specimens from 5 HTLV-I and 5 HTLV-II seropositive US blood donors were obtained from the Abbott Diagnostics Specimen Bank (Abbott Park, IL).

Goat polyclonal antibodies included anti-Friend MuLV (anti-MuLV pAb) and anti-Env (gp69/71) of Rauscher MuLV (anti-Env pAb) (ATCC, VR-1537AS-Gt and VR-1521 respectively). Rat monoclonal antibody to gag p30 MuLV was produced at Abbott Diagnostics (Abbott Park, IL) using hybridoma cells (ATCC, CRL-1912). Mouse monoclonal antibody to Histidine (anti-His Mab) was from Abcam plc, (Cambridge, UK**)**.

### Western blot analysis

Western blot (WB) strips were produced using sucrose gradient purified XMRV (ABI) or recombinant proteins. Virus was lysed with 10 mM Tris-HCl (pH 7.5) buffer containing 150 mM NaCl and 0.5% Triton at 100° C for 10 min. The viral lysate (80 μg/gel) or recombinant proteins (40-80 μg/gel) were separated by electrophoresis on a 4-12% NuPAGE Bis-Tris 2-dimension gel (Invitrogen, Carlsbad, CA) in the presence of sodium dodecylsulfate (SDS). The protein bands on the gel were electrophoretically transferred to a polyvinylidene difluoride (PVDF) membrane (Invitrogen) according to the manufacture's instructions.

WB analysis was performed using WesternBreeze kit reagents (Invitrogen) per the manufacturer's instructions. After blocking, the PVDF membrane was cut into 2 mm strips. WB strips were incubated with macaque sera or human blood donor samples diluted 1:250 (or as specified) overnight at 2-8°C. Goat anti-MuLV pAb and anti-Env pAb were diluted 1:1000 and incubated with WB strips at room temperature for 1 hour. After removal of unbound antibodies, WB strips were incubated with appropriate alkaline phosphatase conjugated secondary antibody for 30 minutes at room temperature. The strips were washed as described and chromogenic substrate solution was added. Goat anti-human IgM and IgG (Southern Biotech, Birmingham, AL) alkaline phosphatase conjugated secondary antibodies were used to individually detect IgM and IgG responses to XMRV infection in the macaque sera.

Competitive inhibition of macaque sera, anti-MuLV pAb or anti-Env pAb binding to WB strips was performed by pre-incubation of the samples with appropriate recombinant XMRV proteins at room temperature for 30 minutes to block specific antibodies. WB analysis was then performed with the pre-absorbed samples as described above.

For comparing antibody responses, WB strips were prepared using recombinant proteins (90 pmole/protein/strip): gp70 (4.4 μg/strip), p15E (4.1 μg/strip) and p30 (3.1 μg/strip). Western blot analysis was then performed with the primate samples diluted 1:200 as described.

### Recombinant proteins

Recombinant proteins derived from XMRV gp70, p15E, p30, p15, p12 and p10 were expressed in *E. coli *or HEK 293 mammalian cells (Invitrogen). For gp70 recombinant protein, a fragment containing amino acids (aa) 1-413 of the surface unit was cloned into an Abbott Laboratories vector selected for use in a mammalian protein expression system (Abbott Bioresearch Center, Worcester, MA) and expressed in DH5α cells (Invitrogen). Purified plasmid DNA was then used to transfect HEK293 cells (Invitrogen). The same sequence was also cloned into CTP:CMP-3-deoxy-D-manno-octulosonate cytidylyl transferase (CKS) expression vector, pJO200 (Abbott Diagnostics), and expressed in XL1-Blue cells (Stratagene, La Jolla, CA) to produce the p70 recombinant protein which lacks glycosylation. For p15E, two versions of the TM protein were expressed. One version (aa 1-201) was cloned into PET expression vector pET-28b (+) (Novagen, Madison, WI) and expressed in *E. coli *BL21 (DE3) cells (Novagen). Another version with 14 aa deletion in the transmembrane region was cloned into pJO200 and expressed in XL1-Blue cells. For p30, the entire CA (aa 1-263) was cloned into both pJO200 and PL expression vector, pKRR826 (Abbott Diagnostics), and expressed in XL1-Blue cells and DH5α cells, respectively. p15 (aa 1-129 of MA), p12 (aa 1-84 of p12), and p10, (aa 1-56 of NC) were cloned into pJO200 and expressed in XL1-Blue cells. All recombinant proteins, except p15E cloned into the pJO200 vector, contained a 6-histidine tag.

Recombinant proteins produced as insoluble inclusion bodies within *E. coli *were solubilized in a solution of 6 M guanidine-HCl, 50 mM Tris-HCl, pH 8.0 and 0.1% β-mercaptoethanol, clarified by centrifugation and purified by Sephacryl S-200 size exclusion chromatography (Pharmacia, Piscataway, NJ). All His tag recombinant proteins were further purified by His-Bind Nickel Affinity Chromatography (Novagen). The pJO200 expressed p15E was purified by Sephacryl S-200 size exclusion chromatography. Purity of all recombinant proteins was estimated at >90% by scanning densitometry.

### Indirect format chemiluminescent immunoassays

The indirect prototype p15E, p70 and p30 antibody assays were developed on the high-throughput (200 tests/hour) and fully automated ARCHITECT^® ^instrument system (Abbott Diagnostics, Dallas, TX). They are two-step chemiluminescent immunoassays (CMIAs) that utilize an indirect (anti-human) assay format (Figure 5A). Capture antigens were prepared by coating the *E. coli*-expressed recombinant proteins (p15E, p70 or p30) onto polystyrene paramagnetic microparticles (Varian, Inc. Palo Alto, CA). Detection conjugate was prepared by labelling a goat anti-human IgG (KPL, Inc. Gaithersburg, MD) with a chemiluminescent compound, N-hydroxysuccinimide ester of 10-sulfopropyl-n-tosyl-n-(2-carboxyethyl)-9-acridinium carboxamide trifluoromethyl sulfonate (SPCP-acridinium, Abbott Diagnostics). The acridinium labeled conjugate was purified with high-performance liquid chromatography (HPLC) using Bio-Sil SEC-250 gel filtration column (Bio-Rad Laboratories, Hercules, CA). In the first step of the assay (Additional file 2, section B1), a serum or plasma sample (10 μL) was mixed with specimen diluent buffer (90 μL) and antigen coated paramagnetic microparticles (50 μL) and incubated at room temperature (r.t.) for 18 minutes. During the incubation, the XMRV-specific antibodies present in the sample were captured on paramagnetic microparticles. Following incubation, the microparticles were washed to remove unbound proteins. In the second step, acridinium-labeled anti-human IgG conjugate (50 μL) was added and incubated at r.t. for 4 minutes. Following an additional wash cycle, alkaline hydrogen peroxide solution was added to release acridinium chemiluminescence signal. The intensity of the chemiluminescence, measured as relative light units (RLU) is proportional to the amount of specific antibody captured by the recombinant proteins.

### Direct format chemiluminescent immunoassays

The prototype direct gp70, p15E and p30 antibody assays are CMIAs developed for the automated ARCHITECT^® ^instrument system (assay format shown in Figure 5B). The capture antigens were prepared by individually coating recombinant proteins (*E. coli*-expressed p15E, p30 or mammalian-expressed gp70) onto polystyrene paramagnetic microparticles (Varian). For direct p15E and p30 CMIAs, detection antigens were prepared by labeling the p15E protein with N^10^-(3-sulfopropyl)-N-(3-sulfopropyl)-acridinium-9-carboxamide pentaflurophenyl ester (SPSP-acridinium, Abbott Diagnostics) and the p30 protein with SPCP-acridinium. Both conjugates were purified with HPLC using Bio-Sil SEC-250 gel filtration column (Bio-Rad Laboratories). The p15E and p30 CMIAs utilized a two-step assay protocol as depicted in Scheme B2 (Additional file 2). In the first step of the assay, a serum or plasma sample (100 μL) was mixed with specimen diluent buffer (50 μL) and p15E or p30 antigen coated paramagnetic microparticles (50 μL) and incubated at r.t. for 18 minutes. During the incubation, the XMRV-specific antibodies present in the sample were captured on paramagnetic microparticles. Following the incubation, the microparticles were washed to remove unbound proteins. In the second step, 50 μl of acridinium-labeled p15E or p30 was added and incubated at r.t. for 4 minutes. Following an additional wash cycle, alkaline hydrogen peroxide solution was added to release acridinium chemiluminescence signal. The intensity of the chemiluminescence, measured as relative light units (RLU) is proportional to the amount of specific antibody captured by the recombinant proteins p15E or p30.

For the direct format gp70 CMIA, avidin-biotin complex (ABC) was used to enhance gp70 conjugate potency. The gp70 protein was labeled with NHS-LC-LC-Biotin (Thermo Fisher Scientific Inc. Rockford, IL) and Streptavidin (Leinco Technologies, Inc. St Louis, Missouri) was labeled with SPCP-acridinium. After purification by HPLC using Bio-Sil SEC-250 gel filtration column (Bio-Rad Laboratories), the purified biotinylated gp70 protein was mixed with purified acridinylated streptavidin at 1.3:1 molar ratio to form the ABC gp70 conjugate. A one-step assay protocol was utilized to provide a longer incubation time for the ABC gp70 conjugate with anti-gp70 antibodies. In the one-step protocol (Additional file 2, section B3) a serum or plasma sample (100 μL), ABC gp70 conjugate (50 μL) and gp70 coated paramagnetic microparticles (50 μL) were combined and incubated at r.t. for 22 minutes. During incubation, anti-gp70 antibodies present in the sample simultaneously bound to the ABC gp70 conjugate and the gp70 antigen-coated paramagnetic microparticles. Following the incubation, the microparticles were washed to remove unbound proteins and ABC gp70 conjugate. Chemiluminescent signal measurement was performed as described above.

## Competing interests

XQ, PS, KCL, BT, SD, GS and JH are employees of Abbott Diagnostics.

RHS, EAK and JDG: patents, Abbott Diagnostics. RHS consulting: Abbott Diagnostics

## Authors' contributions

XQ carried out characterization of antibody responses in XMRV-infected macaques and developed XMRV prototype assays. PS, KCL, BT and JDG developed XMRV recombinant proteins. FV established XMRV infection in the non-human primate model. PS participated in sample testing. JH, SD, GS, FV, RHS and EAK conceived and designed the study. XQ, JH and PS wrote the manuscript. All authors read and approved the final manuscript.

## Supplementary Material

Additional file 1 **WB analysis of CMIA reactive blood donor samples**. A1, p15E CMIA reactive blood donor, A2, gp70 CMIA reactive blood donors and A3 p30 CMIA reactive blood donors were analyzed by WB with native XMRV viral proteins (4 μg/strip). A4, gp70 CMIA reactive blood donors were analyzed by WB with mammalian expressed recombinant gp70 WB. Primate bleed (RIl-10) and anti-MuLV pAb were used as positive controls. Click here for file

Additional file 2 **Schematic diagrams of XMRV CMIA assay formats**. B1, Indirect (anti-human) 2-step assay format. B2, Direct (double antigen sandwich) 2-step assay format. B3, Direct (double antigen sandwich) 1-step assay format. rAgs = recombinant antigens; hv = chemiluminescent signal. Click here for file
