# Supplementary figures and images for: Characterization of antibodies elicited by XMRV infection and development of immunoassays useful for epidemiologic studies
Source: Retrovirology. 2010 Aug 17;7:68. doi: 10.1186/1742-4690-7-68 (PMC2931451; doi:10.1186/1742-4690-7-68)

A1

A2

A3

A4

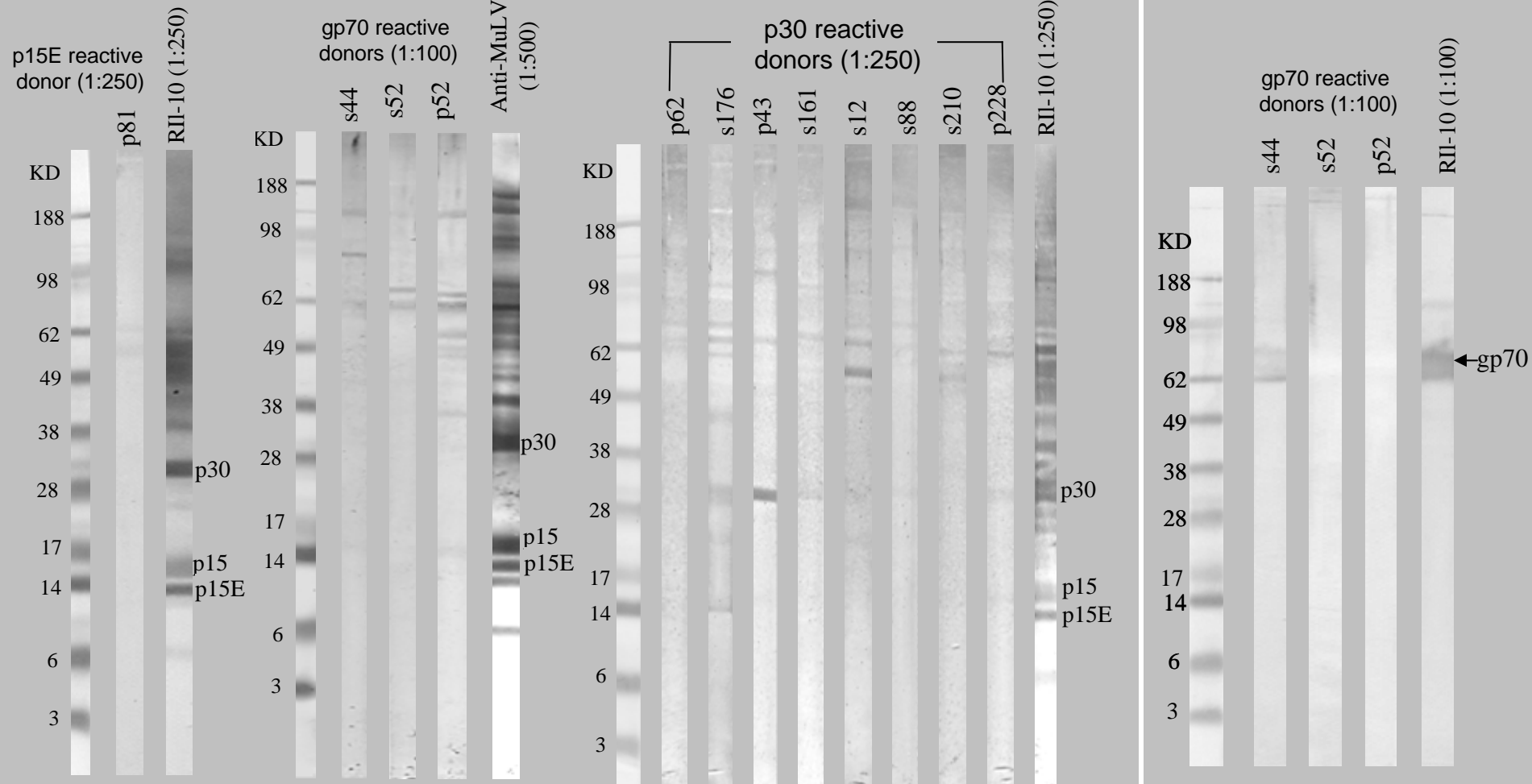

XMRV Viral lysate WB (4.0 ug/strip)

Recombinant gp70 WB  
(2.0 ug/strip)

Supplement: Additional file 1 — WB analysis of CMIA reactive blood donor samples. A1, p15E CMIA reactive blood donor, A2, gp70 CMIA reactive blood donors and A3 p30 CMIA reactive blood donors were analyzed by WB with native XMRV viral proteins (4 μg/strip). A4, gp70 CMIA reactive blood donors were analyzed by WB with mammalian expressed recombinant gp70 WB. Primate bleed (RIl-10) and anti-MuLV pAb were used as positive controls. [file 1742-4690-7-68-S1.PDF]
