# Supplementary material for: Characterization of antibodies elicited by XMRV infection and development of immunoassays useful for epidemiologic studies
Source: Retrovirology. 2010 Aug 17;7:68. doi: 10.1186/1742-4690-7-68 (PMC2931451; doi:10.1186/1742-4690-7-68)
Supplement: Additional file 2 — Schematic diagrams of XMRV CMIA assay formats. B1, Indirect (anti-human) 2-step assay format. B2, Direct (double antigen sandwich) 2-step assay format. B3, Direct (double antigen sandwich) 1-step assay format. rAgs = recombinant antigens; hv = chemiluminescent signal. [file 1742-4690-7-68-S2.PDF]

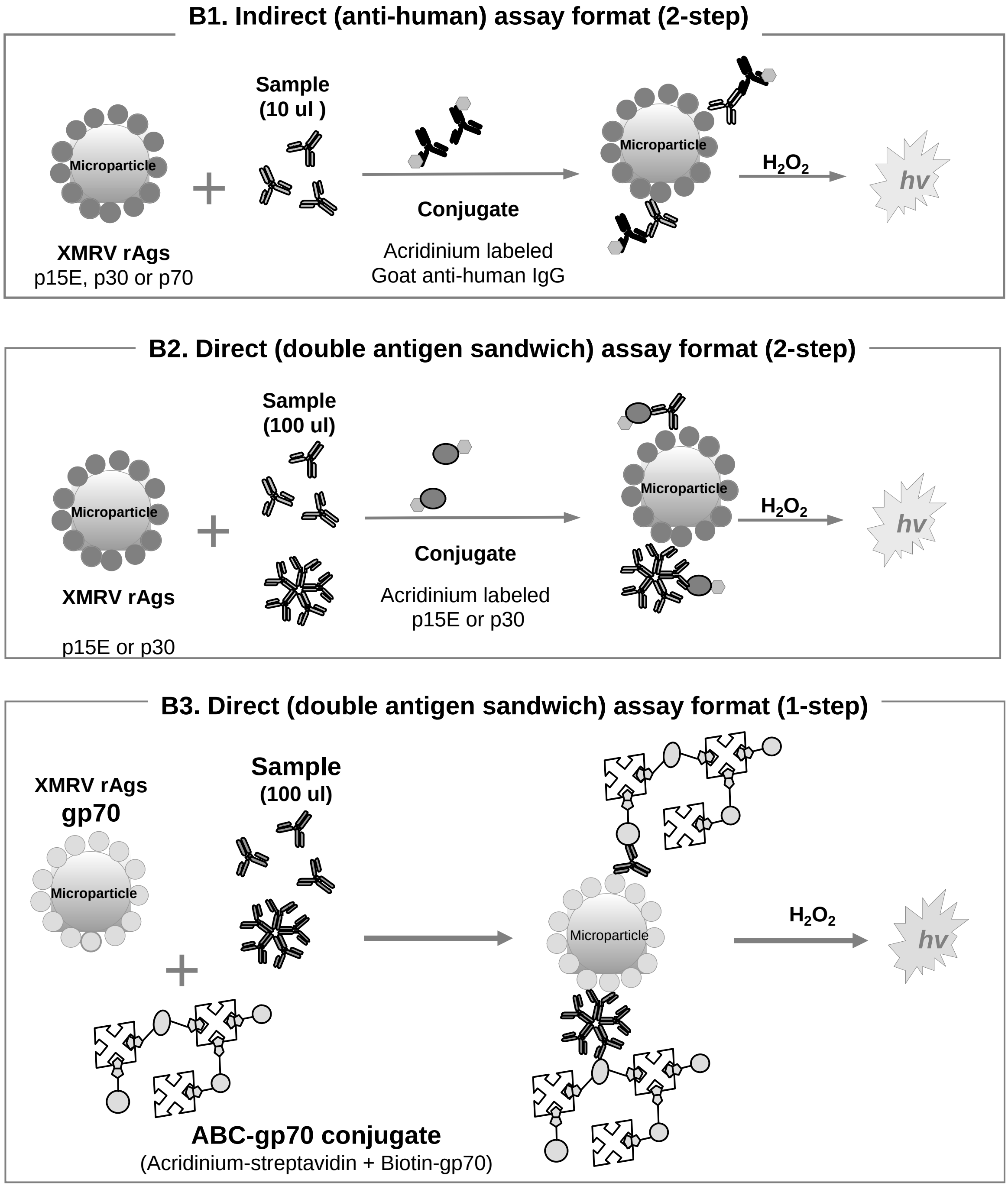
B1. Indirect (anti-human) assay format (2-step)
Microparticle
XMRV rAgs
p15E, p30 or p70
Sample
(10 ul)
Conjugate
Acridinium labeled
Goat anti-human IgG
Microparticle
H_2O_2
hv
B2. Direct (double antigen sandwich) assay format (2-step)
Microparticle
XMRV rAgs
p15E or p30
Sample
(100 ul)
Conjugate
Acridinium labeled
p15E or p30
Microparticle
H_2O_2
hv
B3. Direct (double antigen sandwich) assay format (1-step)
XMRV rAgs
gp70
Microparticle
Sample
(100 ul)
ABC-gp70 conjugate
(Acridinium-streptavidin + Biotin-gp70)
Microparticle
H_2O_2
hv
